# Evaluating the antibacterial effect of cobalt nanoparticles against multi-drug resistant pathogens

**DOI:** 10.25122/jml-2021-0270

**Published:** 2021

**Authors:** Abeer Abdulridha Abass, Wasna a Mohammed Abdulridha, Warood Kream Alaarage, Noor Hassan Abdulrudha, Julfikar Haider

**Affiliations:** 1. Basic Science Department, College of Dentistry, University of Kufa, Najaf, Iraq; 2. Department of Engineering, Manchester Metropolitan University, Manchester, United Kingdom

**Keywords:** cobalt nanoparticle, nanomedicine, pathogenic bacteria, antimicrobial activity, antibiotics, AI – Activity index, Co NPs – Cobalt nanoparticles, MH – Muller-Hinton agar medium, MDR – Multidrug-resistant, SEM – Scanning Electron Microscope, XRD – X-Ray diffraction

## Abstract

This study aimed to estimate the effect of cobalt nanoparticles (Co NPs) with different concentrations against multidrug-resistant (MDR) pathogenic bacteria. Three isolates of *Staphylococcus aureus* (gram-positive), *Proteus spp*. (gram-negative), and *Escherichia* *coli* (gram-negative) bacteria were extracted from various clinical examples utilizing routine methods on bacteriological culture media. The antibacterial sensitivity of commercial antibiotics such as Ciprofloxacin, Cefotaxime, Gentamycin, and Amoxicillin was broken down on a Muller Hinton agar plate and evaluated using the disk diffusion method. The study results demonstrated the antibacterial effect of the Co NPs against the bacterial isolates with three different concentrations utilized in the study. The results indicated that the Co NPs showed the highest antibacterial activity when utilizing 100 μg/ml against *Escherichia coli* followed by *Proteus spp* and *Staphylococcus aureus* with zones of inhibition measured as 22.2±0.1 mm, 20.3±0.15 mm, and 15.8±0.1 mm; respectively. Co NPs at a 100 μg/mL concentration showed higher inhibition zones than several common antibiotics except for Ciprofloxacin, which demonstrated better antibacterial activity against the bacterial isolates employed in this study. Scanning Electron Microscope (SEM)and X-Ray diffraction (XRD)studies confirmed that Cobalt nanoparticles (Co NPs) were synthesized from cobalt sulphate solution with a size ranging from 40 nm to 60 nm. The nanoparticles showed a crystalline structure with a round shape and smooth surface. The antibacterial resistance of Co NPs against three common bacteria such as *Staphylococcus aureus*, *Proteus spp*, and *Escherichia coli* was assessed in this study. The optimum concentration of the Co NPs was identified as 100 μg/ml, which could provide a similar or higher antibacterial effect.

## Introduction

Diminishing antimicrobial resistance is quickly becoming a worldwide concern with a fast increase in multidrug-resistant (MDR) bacteria [1]. *Escherichia coli*, *Staphylococcus aureus*, and *Klebsiella pneumonia* are the most well-known MDR bacteria related to nosocomial infections [2–3]. In recent years, the utilization of nanotechnology and the blend and production of nanoparticles (NPs) have brought new expectations for the fight against MDR bacteria [4]. In addition, nanoscale materials have appeared as the new antimicrobial agents. Some classes of antimicrobial NPs and nanosized carriers for antibiotics delivery have demonstrated their efficiency in treating infectious diseases *in vitro*, including the antimicrobial-resistant ones [5]. The rapid development of nanotechnology has provided several materials for biomedical applications, including those used as anti-microbes [4]. Modern drug delivery techniques operate on the highly beneficial principle of site-specific or targeted therapy, and the use of nanoparticles in various medical applications has allowed for drug therapy and various applications related to visualization, sensing, and gene delivery [6]. NPs have attracted extraordinary interest in their improvement as potential antibacterial drugs [7–8]. Over the most recent years, many studies have investigated the structure and chemical behavior of some metals and metal oxides to discover new drugs with antibacterial capabilities. Among them, Ag, Au, Co, TiO2, ZnO, CuO, Fe_2_O_3_ etc, have proven their ability to act as an antibiotic [4–9]. Cobalt complex showed promises as a good drug of choice to manage bacterial, fungal, or amoebal diseases as outlined in recent publications [10–13]. Over the past years, several studies have been carried out on the antibacterial activity of Co NPs [14–18]. Igwe *et al.* concluded that Co NPs with hexagonal shapes and sizes ranging from 20–49 nm could be employed for treating infections by inhibiting the growth of *Escherichia coli*, *Klebsiella pneumonia*, *Staphylococcus aureus*, and *Streptococcus pyogene* [14]. Kharade *et al.* [15] demonstrated excellent antibacterial activity of green synthesized Co NPs with an average size of 20.88 nm using Hibiscus cannabinus leaf extract against *Bacillus substilis* and *Escherichia coli*. Co NP was synthesized using Raphanussativus var. longipinnatus leaf extract showed effective antibacterial activity against gram-negative bacteria such as Pseudomonas putida and *Klebsiella pneumonia* [16]. In this case, the NPs was characterized as a spherical shape of slightly bigger size of 80 nm. Raza *et al.* [17] presented the results of the antibacterial performance of three bacteria, *E. coli*, *P. aeruginosa*, and *B. subtilis*, at different concentrations of the Co NPs 1 mg/ml, 50 mg/ml, and 100 mg/ml, respectively. It was concluded that the best performance was obtained at the highest concentration. Shahzadi *et al.* [18] tested the performance of CoNPs with an average particle size of 27.42 nm on antimicrobial activity. Co NPs showed lower activity against gram-positive bacteria *B. subtilis* (inhibition zone diameter: 42.18 mm) compared to the gram-negative *E. coli* 51.83 mm. However, compared with a reference antibacterial drug Rifampicin, both bacteria showed lower inhibition zone diameters. In a more recent study, Gupta *et al.* [19] reported that Co NPs demonstrated better antibacterial efficacy than their bulk form. Furthermore, the Co NPs were effective even at lower concentrations (0.125 μg/ml) against *S. aureus* and *E. coli* and showed better efficacy than standard antibiotics. Satpathy G. and Manikandan E. [20] reported that cobalt nanoparticles have a sensitive antiseptic effect for the gram-negative *Escherichia coli* strains and present the results of the inhibition zone (mm) of different concentrations of Co NPs of about 18, 20, 25, 27 in diameter for 5 μg/ml 15 μg/ml, 25 μg/ml, and 35 μg/ml of Co NPs concentrations, respectively. Anwar *et al.* [21] reported that Co NPs prepared by different techniques (utilizing hydrothermal and ultrasonication) were used as novel nanotherapeutics against *Acanthamoeba castellanii*. At present, the limited information available in the literature about the effectiveness of the cobalt nanoparticle (Co NPs) as an antibacterial agent on varieties of bacteria demands further investigation. Although several reports are available on killing bacteria with Co or cobalt complex NPs, to the best of the author’s knowledge, no studies have determined the antibacterial effect of Co NPs against all three selected bacteria in this study. Furthermore, studies related to comparative assessment on the antibacterial effects between the Co NPs and commonly available antibiotics are still lacking. Therefore, the present study was conducted to determine the physical properties and to estimate the antimicrobial activity of the Co NPs against three multi-drug resistant bacteria.

## Material and Methods

**Figure 1.**
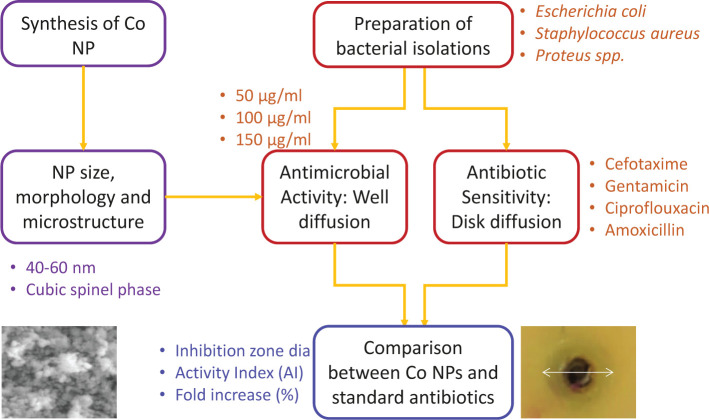
Methodology adopted for evaluating the antibacterial effect of Co NPs.

The study design to synthesize Co NPs and assess their antibacterial effect is presented in Figure 1.

### Preparation and characterization of Cobalt Nanoparticles (Co NPs)

Hydrazine monohydrate and cobalt sulfate heptahydrate (CoSO_4_.7H_2_O) were utilized as the precursors for synthesizing Co NPs [22]. A 0.2 M solution of sodium citrate dihydrate was added to 10 ml of 0.1 M aqueous cobalt sulfate solution. The solution was kept up at a foreordained temperature and permitted to respond for 60 min to 120 min. Afterward, the solution was centrifuged at 4000 rpm for 1hr; the suspensions were taken out and washed a few times with distilled water and dried utilizing a vacuum dryer at a temperature of 80°C. The nanoparticles on a glass slide were placed in a scanning electron microscope (SEM) to observe the particle morphology (Inspect S50, FEI company, Netherlands) at an accelerating voltage of 12.5 kV. The phase structure and orientation of the Co NPs were determined by X-ray diffraction (XRD) technique using a Shimadzu-XRD 6000 (Shimadzu Company, Japan) diffractometer with a Bragg Brentano geometry and employing a CuKα source (40 kV, 30 mA) at an incident angle of 2°. The average crystallite dimensions were estimated by the Scherrer formula [23].



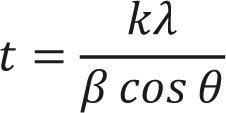



Where k is the shape factor (usually 0.9), λ is the X-ray wavelength, θ is the Bragg diffraction angle, β is the full width at half maximum (FWHM) in radians.

### Bacterial Isolation and Identification

During the study, three isolates of *Escherichia coli*, *Staphylococcus aureus*, and *Proteus spp*. were isolated from different clinical samples, and bacteria were identified using blood agar, MacConkeyagar, and Nutrient agar (Difco, USA). The plates were incubated for 24h at 37°C. The classical method was utilized to identify bacteria by comparing with systematic Bacteriology Bergey’s Manual.

### Antimicrobial Activity Measurements of Co NPs

An agar well diffusion method was used to assess the antibacterial activities of Co NPs against the isolates of *Escherichia coli*, *Staphylococcus aureus*, and *Proteus spp*. [19–24]. All bacteria were suspended in sterile water and diluted to 1×10^8^CFU/ml. The suspension was spread over Mueller Hinton’s agar by sterile cotton swab, and after 15 min, wells (8mm) were cut into the agar using a sterilized cork borer. The lower ends of the wells were closed with molten agar to prevent any leakage of the tested nanomaterial. An equal volume of 100 μl Co NPs was taken from different concentrations of the suspensions 50, 100, 150 μg/ml using a micropipette and separately poured onto the wells. The Petri plates were incubated at 37°C for 24h, and the inhibition zones were measured for each concentration and microbes. The inhibition zone is defined as the clear zone created around the wells by the antibacterial action of the Co NPs. Negative controls were set using sterile water, and the positive control using antibiotics. As expected, no inhibition zone was observed in the case of the negative control. The antibacterial activity was assessed by determining the diameters of inhibition zones of the tested bacteria according to National Committee for Clinical Laboratories Standard rules [25]. The greater the inhibition zone, the greater the antibacterial activity. All measurements were executed three times to obtain an average result.

### Antibiotic Sensitivity Testing

The disk diffusion method on Muller-Hinton agar medium (MH) (Oxoid, UK) was performed to assess the sensitivity of *Staphylococcus aureus*, *Escherichia coli*, and *Proteus spp*. isolates against standard antibiotic disks of 8 mm, including Cefotaxime, Gentamicin, Ciprofloxacin, and Amoxicillin (Himedia, India). The isolates were suspended in sterile water and diluted according to MacFarland microbial suspension, which approximately contained 1×10^8^ CFU/ml. The cultures were incubated at 37°C for 18 hr, according to Kirby-Baur. The zones of inhibition were determined by the National Committee for Clinical Laboratories Standard rules [25]. All measurements were executed three times to obtain an average result. Activity Index (AI) and Fold Increase were calculated based on the inhibition zone diameters using Equation 2 and Equation 3 to compare the performance of Co nanoparticles compared to standard antibiotics [19–24].



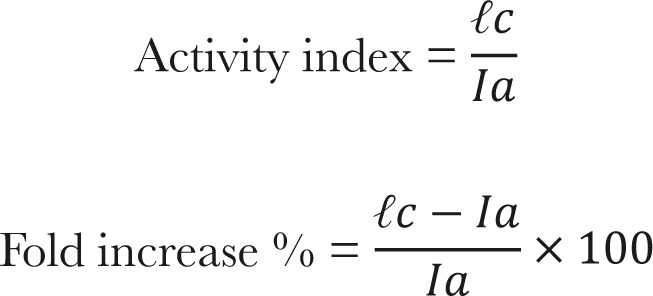



Where Ic is the inhibition zone diameter of Co NPs and Ia is the inhibition zone diameter of antibiotics.

### Statistical analysis

A statistical design of experiments was used to study the antibacterial effect of Co NPs at different concentrations, the inhibition zone diameters of cobalt nanoparticles compared with the antibiotics, and the activity index (AI) of cobalt nanoparticles compared with the antibiotics.

## Results

### Structural characteristics of Co NPs

**Figure 2.**
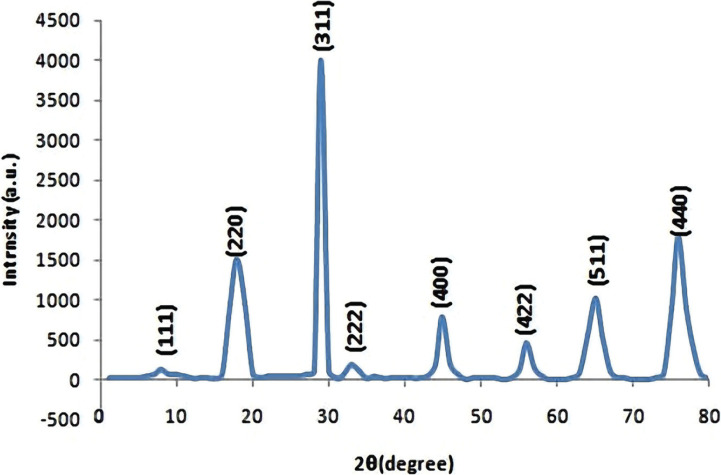
XRD pattern of synthesized CoNPs.

The Co NPs crystalline structure was established by XRD, as revealed in Figure 2. The XRD patterns showed a diffraction line assigned to the pure cubic spinel phase. Well-developed peaks corresponded to 111, 220, 311, 222, 400, 422, 511, and 440 crystal planes. All measured XRD peaks match well with the standard patterns of Co. The sharp peaks confirmed the crystallinity of the Co NPs. It was also concluded that the Co NPs were pure as no peaks related to other materials were observed [26]. The particle size was estimated in the range of 50 nm to 55 nm using the Scherrer formula.

**Figure 3.**
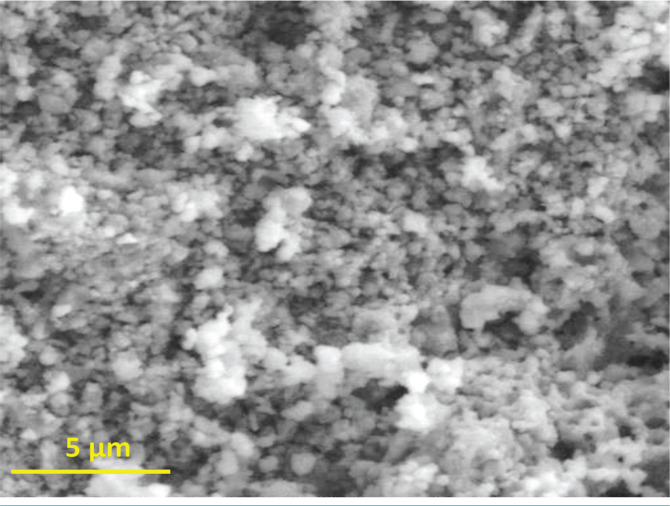
SEM Image of Co nanoparticles.

The morphological image of the CoNPs shown in Figure 3, was taken by a scanning electron microscope (SEM). The SEM image confirmed the formation of nanosized crystallites with spherical shapes. The average grain size was found to be in the range of 40 nm to 60 nm when measured using the linear intercept method. Furthermore, smaller particles were amalgamated to form clusters of larger size. Other researchers made similar observations about the Co NPs [26–27]. In general, the particle shape and size were uniform with a smooth surface, which could be related to contact with the bacteria to demonstrate increased antibacterial activities [27–28].

### Assessment of Antibacterial effect: Well diffusion

**Figure 4.**
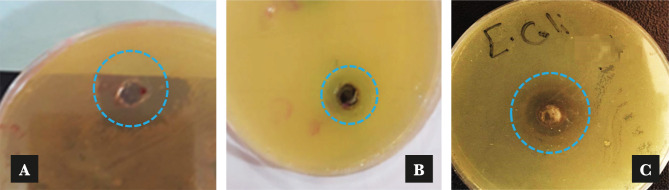
Inhibition zones of bacterial isolates with 100 μg/ml concentration of Co NPs: (a) Staphylococcus aureus, (b) Proteus spp. and (c) Escherichia coli (not to be scaled).

Table 1 presents the means and standard deviations of inhibition zone diameters at three different Co NPs concentrations. The results of the well diffusion method exhibited the highest antibacterial activity against *Escherichia coli* followed by *Proteus spp*. and *Staphylococcus aureus* indicated by the zones of inhibition reaching 22.2±0.1 mm, 20.3±0.15 mm, and 15.8±0.1 mm, respectively when a Co NPs concentration of 100 μg/ml was used. The antibacterial effect of Co NPs at different concentrations was defined by a clear zone around the wells. While the lowest inhibition zone was recorded against *Proteus spp*. with a diameter of 10.26±0.15 mm at 50 μg/ml of Co NPs. However, the inhibition zones were 11.5±0.1 mm and 13.23±0.15 mm against each *Staphylococcus aureus* and *Escherichia coli*, when the Co NPs concentration was 50 μg/ml. While 150 μg/ml of Co NPs recorded lower effects than the 100 μg/ml concentration having a lowest mean inhibition zone of 12.26±0.15 mm against the *Proteus spp*. Figure 4 presents example images of inhibition zones for different bacteria. These results agreed with Satpathy G., Manikandan E. [20], and Moradpoor *et al.* [29], who reported that the Co NPs showed antibacterial activity when exposed to pathogenic activity bacteria using the wells diffusion technique.

**Table 1.** Antibacterial effects of the cobalt nanoparticles using well diffusion method.

Bacterial type	Mean±SD of inhibition zone diameters (mm) at different Co NP concentrations		
	**50 μg/ml**	**100 μg/ml**	**150 μg/ml**
Proteus spp.	10.26±0.15	20.3±0.15	12.26±0.15
Staphylococcus aureus	11.5±0.1	15.8±0.1	13.2±0.1
Escherichia coli	13.23±0.15	22.2±0.1	15.16±0.5

### Assessing the effect of antibiotic: Disk diffusion

**Figure 5.**
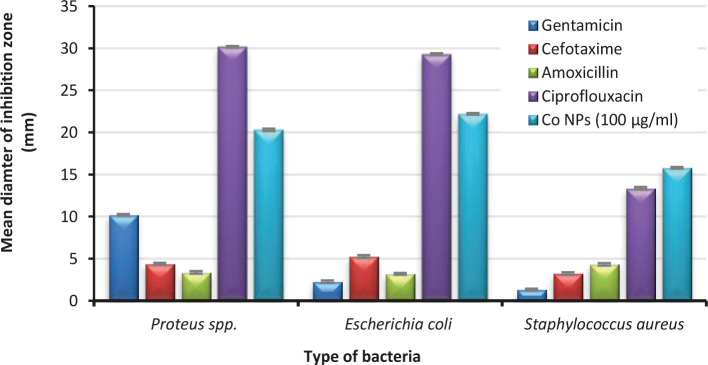
Mean of inhibition zone diameters of 100 μg/ml of cobalt nanoparticle compared with the antibiotics.

Figure 5 presents the diameters of the inhibition zone when anti-microbials sensitivity was measured against the *Staphylococcus aureus*, *Escherichia coli*, and *Proteus spp*. isolates controlled by a disk diffusion method. All the isolates demonstrated a high resistant rate (inhibition zone≤5 mm) to the most antibiotics utilized in this study, including Gentamicin, Ceftriaxone, and Amoxicillin except for Ciprofloxacin, which recorded the zones of inhibition extended to 30.17±0.1mm, 29.30±0.1 mm, and 13.37±0.15 mm against *Proteus spp*., *Escherichia coli*, and *Staphylococcus aureus* respectively.

**Figure 6.**
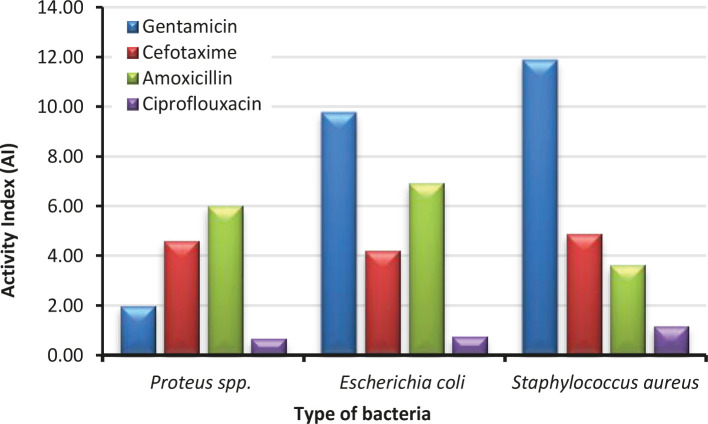
Activity Index (AI) of 100 μg/ml of cobalt nanoparticle compared with the antibiotics.

Figure 6 presents the Activity Index (AI) of Co NPs. AI is a relative measure of the effectiveness of one antimicrobial drug compared to another antibiotic drug. In general, if the AI value is greater than unity, the tested material (Co NPs) would be better than an antibiotic drug against a particular strain of bacteria [19]. The AIs were calculated for all four standard antibiotics, Gentamicin, Cefotaxime, Amoxicillin, and Ciprofloxacin, against the three selected bacteria in this study. In the case of *Proteus spp*. AI values of Co NPs compared to Gentamicin 1.99, Cefotaxime 4.61, and Amoxicillin 6.02 were higher than 1, indicating its better antibacterial effect. However, an AI value 0.67 lower than 1 was found for Co NP compared to Ciprofloxacin. This result led to believe that Co NPs might not be better than all antibiotics. In the case of *Escherichia coli*, the AI values of Co NPs compared to Gentamicin 9.78, Cefotaxime 4.21, and Amoxicillin 6.94 were higher than 1 except Ciprofloxacin 0.76. In the case of *Staphylococcus aureus*, the AI values of Co NPs compared to Gentamicin 11.88, Cefotaxime 4.89, Amoxicillin 3.65, and Ciprofloxacin 1.18 were higher than 1. In summary, Co NPs at a concentration of 100 μg/ml showed better bacteria-killing ability against all three strains than all four antibiotics except Ciprofloxacin against *Proteus spp*. and *Escherichia coli*.

**Figure 7.**
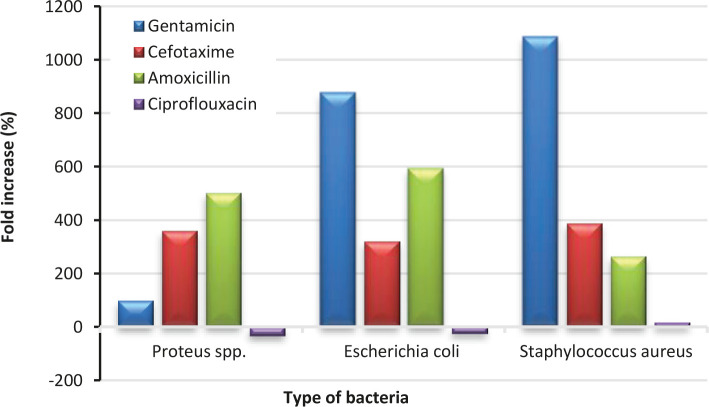
Fold increase (%) of 100 μg/ml of cobalt nanoparticle compared with the antibiotics.

Fold increase (%) of Co nanoparticles also represented another relative measure of its antibacterial actions compared to the standard antibiotics used, as shown in Figure 7. A positive fold increase value indicated a better efficacy of the Co NP than the tested antibiotics and vice versa. Again, similar conclusions could be drawn from the results as determined by the AI results. Negative fold increases of Co NPs with respect to Ciprofloxacin against *Proteus spp* -32.71% and *Escherichia coli* -24.23% revealed slightly poorer antibacterial action than this particular antibiotic. However, significantly higher positive fold increases compared to the other three antibiotics demonstrate the superior antibacterial performance of the Co NPs.

## Discussions

**Figure 8.**
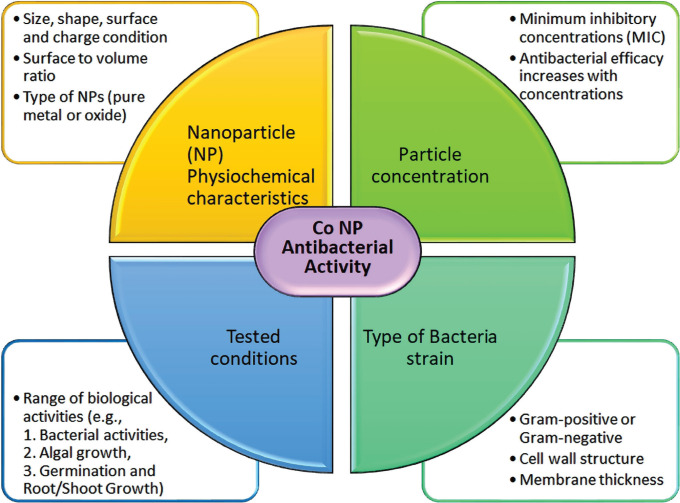
Factor affecting Co NPs effectiveness in antibacterial action.

Variations were noticed in the inhibition zone diameters between this study and other studies reported in the literature. In general, for a relatively high concentration of Co NPs the inhibition zone diameters in this study were lower than the other reported values. For example, Satpathy and Manikanda evaluated the prophylactic activity of Cobalt nanoparticles towards isolated *Escherichia coli* with the infusion prepared at four concentrations from 5–35 μg/ml [20]. The optimum concentration was identified as 35 μg/ml, which generated an inhibition zone diameter of 27 mm. However, in this study, for the same bacteria, the inhibition zone diameter was 13 mm even at a slightly higher concentration of 50 μg/ml. This could be due to the difference in size and characteristics of the Co NPs used and their interaction with the bacteria cells. The Co NPs played a role as a potential antiseptic to control the infections by other bacteria such as *B. Subtilis* and *Pseudomonas Sp*. [10–27]. It was also suggested that Co NPs were biosafe when cell cytotoxicity tests produced minimal damage to human cells at a nanoparticle concentration of 100 μg/ml [13]. Other than Co or cobalt oxide, nanocomposite made of graphene-cobalt oxide also showed potential for antibacterial activity [30]. Literature studies reported that the inhibition zone diameter continuously increased with the increase in NP concentration [19–27]. However, for all three bacteria in this study, the inhibition zone diameters decreased at 150 μg/ml when compared to the concentration of 100 μg/ml. The exact reason for this decrease was not entirely clear. Therefore, further studies are required to explore the antibacterial effect beyond a Co NPs concentration of 150 μg/ml. A similar observation was also made by Gupta *et al.* [19] that the diameter of inhibition zones increased up to Co NPs concentration of 128.0 μg/ml against *S. aureus* and *E. coli*. and the inhibition zone started to shrink beyond this concentration. The authors reasoned that the nanoparticles might start to agglomerate beyond an optimum concentration and gradually lose their ability to penetrate the bacteria cell. It was reported that the size of the nanoparticles could play an important role in the antibacterial activity as the surface-to-volume ratio increases with a reduction in the size of the nanoparticles [9]. Kong *et al.* [26] evaluated the effects of two different Co NPs sizes on the antibacterial characteristics. Group A nanoparticle had a size ranging between 10 nm to 30 nm with some particles larger than 50 nm, whereas Group B mainly had a range between 80 nm to 150 nm with many over 200 nm. In particular, it was confirmed that there was a clear indication that smaller CoNPs showed a higher 1.2-to-1.5-fold inhibitory effect compared to the larger nanoparticle group with the tested conditions. It was hypothesized that the physical characteristics of the particles were more impactful compared to the antibacterial action caused by the ions released by the CoNPs. In general, the effect of Co NPs depends on the NP’s physical & chemical characteristics, particle concentration, the type of bacteria strains, and the tested conditions, Figure 8 [26–28].

**Figure 9.**
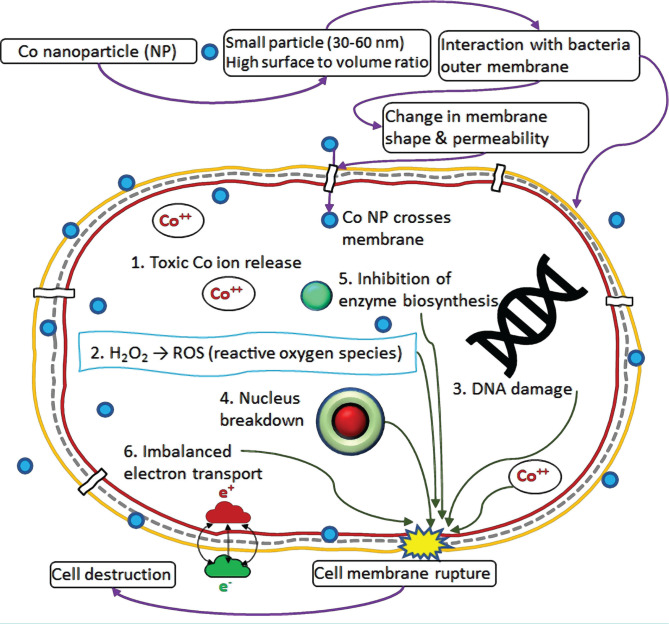
Possible cell death mechanism of bacteria in contact with Co nanoparticles

It was clear from Table 1 that the inhibition zones for the gram-negative bacteria (*Proteus spp*. and *Escherichia coli*) were bigger than that of the gram-positive bacteria (*Staphylococcus aureus*). Other researchers noted similar observations for Co NPs [26–31]. This behavior could generally happen due to the difference in the bacterial cell wall structure and/or size, shape, surface, and charging states of the metallic nanoparticles used. This could be perceived because the cell walls of the gram-negative bacteria (*Proteus spp*. and *Escherichia coli*) were comprised of thin layers of peptidoglycan 8 nm under the outer membrane lipopolysaccharides (1–3 μm thick). On the other hand, the gram-positive bacteria (*Staphylococcus aureus*) possess a thicker peptidoglycan 80 nm layer with a porous structure. This difference in cell structure makes the gram-negative bacteria more susceptible to cell destruction [28–32]. Further details about the gram-positive and gram-negative bacteria were provided by Hoseinzadeh *et al.* [7]. Other physicochemical characteristics of the nanoparticles used and their interaction with the cell wall can play an important role in showing strong antibacterial action against gram-negative bacteria. The positively charged Co ions could be attracted by the cell of the gram-negative bacteria and led to inhibition of different biological processes. Furthermore, a greater tendency of the Co NPs to agglomerate might reduce its antibacterial action against the gram-positive *Staphylococcus aureus* [33]. However, for the cases of Co oxide and other oxide NPs, evidence of the opposite trend was also noticed [24–27]. The higher effectiveness of the Co oxide NPs against gram-positive bacteria could be related to an enhanced cell wall permeability resulting from the interactions with the nanoparticles [12]. At present, there is still limited understanding on the exact mechanism of the antibacterial action of the metallic nanoparticles. However, based on different explanations provided in the literature [3, 11, 13, 32–35], the antibacterial actions of the Co NPs could be summarized as in Figure 9. First, the small Co NPs with a high surface-to-volume ratio interacted with the bacteria’s outer membrane and caused a change in its shape and permeability. This higher permeability allowed the NPs to enter the cell. Toxic Co ions were then released by the nanoparticles and induced the synthesis of highly reactive oxygen species and cellular oxidative stress [36]. This could cause damage to the DNA [37], nucleus breakdown, and imbalance in electron transport across the cell wall. Furthermore, the interaction of Co NPs with thiol groups (-SH) of enzymes in bacteria causing inactivation and death of pathogens could be a potential mechanism for the antibacterial action [27]. The existence of metal ions on the bacterial cell surface is facilitated by the thiol groups (-SH) of proteins. The produced proteins with the carried nutrients penetrated the cell membrane, wherein the inactivation of proteins was initiated by the NPs, thus causing the bacterial demise [11]. Finally, Co NPs showed remarkable inhibition effects against the growth of *E. coli* and *S. aureus*; they can be deployed for treating infectious diseases that occurred by these pathogenic organisms.

One of the objectives of the present study was to estimate the antibiotic resistance of the clinical isolates [38]. Bacteria developed different methods to be resistant against anti-microbials. For example, resistance to β lactam antibiotics (Ceftriaxone and Amoxicillin) was essentially caused by the production of β-lactamases, which was noticed among all the isolates in the present study. The β- lactamases enzyme plays a major role in the resistance to β-lactam antibiotics. Also, *Staphylococcus aureus* becomes resistant to the β-lactam drug by decreasing the permeation through the outer membrane, which reduces the affinity for penicillin-binding protein [38–39]. Furthermore, some aminoglycosides and Ciprofloxacin might show *in vitro* action against *Escherichia coli* and *Proteus spp*. isolates, leaving a long way off the β-lactam antibiotics. It was interesting to note that although Ciprofloxacin was the best among the antibiotics used, again, similar to Co NPs, it showed better action against the gram-negative bacteria than the gram-positive one. Poor resistance of gentamicin against *Escherichia coli* 2.27±0.15 mm and *Staphylococcus aureus* 1.33±0.1 mm might be due to the production of various active enzymes, including aminoglycoside modifying enzymes (AME) which could damage the power of antibiotics [40]. Several fluoroquinolone antibiotics, such as ciprofloxacin, displayed a high action of fluoroquinolones to prevent bacterial growth by inhibiting DNA replication. More lately, researchers have recognized that plasmid-mediated horizontally transferable genes (Qnr) are capable of protecting DNA gyrase from quinolones [41]. Varaprasad *et al.* evaluated the antibacterial performance of green synthesized Co NPs (48 nm) using the disc diffusion with a concentration of 50 μg/ml against human pathogenic gram-negative bacteria (*S. dysenteriae* and *E. faecalis*) and compared to a standard antibiotic [42]. The inhibition zone diameters for these two bacteria (14 mm and 13 mm, respectively) were slightly lower than Ciprofloxacin (15 mm), indicating that Co NPs demonstrated effective antibacterial activity on the pathogenic bacteria. Gupta *et al.* studied the antibacterial activity of Co NPs from very low (0.125 μg/ml) to high (128.0 μg/ml) concentrations against *S. aureus* and *E. coli* [19]. The results clearly indicated better antibacterial action of Co NPs compared to the bulk Co and gentamicin at all concentrations but slightly poorer than Oxytetracycline. This result agreed well with the present study. Hafeez *et al.* also found that Co oxide nanoparticles showed better antibacterial action against two gram-positive bacteria, Bacillus subtilis and Bacillus licheniformis, compared to bacitracin antibiotics [27]. Al-Tamimi [34] showed high growth inhibition against *Staphylococcus aureus* and *Escherichia coli* with Co_3_O_4_ NPs than amoxicillin at 100 μg/ml concentration. However, not many antibiotics were tested in the above studies, unlike four antibiotics in this study. 

It was clear that Co NPS were more effective against the two gram-negative bacteria compared to the one gram-positive bacteria used in this study. Further studies could explore its effectiveness on other gram-positive bacteria to identify a clear trend. This finding also indicated that Co NPs alone are not equally effective against all types of bacteria. Therefore, the applications of multiple nanoparticles [43] or a combination of standard antibiotics and Co NPs [44–45] can be further studied. The efficiency of Co NPs synthesized by a green route can be compared as well [10]. With all these development opportunities, Co NPs can play a vital role against antibacterial infections for therapeutical applications and create a revolution in the healthcare sector with applications including medical devices such as catheters, wound care, dental hygiene, and antibacterial soaps.

Furthermore, it is envisaged that the applications of Co NPs can go far beyond the healthcare sector, such as the garments industry, food and drink industry, care homes, water purification, air purifications, manufacturing industry, chemicals, and pharmaceuticals industry etc with Co NPs based products in the forms of a solution, paste, thin coating, or solid nanocomposite material. In real-life scenarios, there could be more than one MDR bacteria present in the same location. This study demonstrated that the Co NPs were equally effective against some common MDR gram-positive and gram-negative bacteria. Therefore, in the future, Co NPs give hope of developing new therapeutic medicine to replace the less effective traditional standard antibiotic.

## Conclusion

In summary, SEM and XRD studies confirmed that Cobalt nanoparticles (Co NPs) were synthesized from cobalt sulphate solution with a size ranging from 40 nm to 60 nm. The nanoparticles showed a crystalline structure with a round shape and smooth surface. The antibacterial resistance of Co NPs against three common bacteria such as *Staphylococcus aureus*, *Proteus spp*., and *Escherichia coli* was assessed in this study. The results demonstrated that the Co NPs possessed strong antibacterial activity during the *in vitro* examination and may help kill multidrug-resistant (MDR) bacteria and be suitable for treating disease. The Co NPs were effective for both gram-positive and gram-negative bacteria. The optimum concentration of the Co NPs was identified as 100 μg/ml, which could provide a similar or higher antibacterial effect than several standard antibiotics available in the market. Moreover, this study suggested that the CoNPs can be used in medical instrumentation, antibiotics disinfection, and detergents. The prepared CoNPs showed amazing antibacterial activity against *Staphylococcus aureus* (gram-positive), *Proteus spp*. (gram-negative) and *Escherichia coli* (gram-negative) bacteria. The results obtained confirm that green synthesized CoNPs using Hibiscus cannabinus leaf extract will bring a promising application in the field of medicine.

## Acknowledgments

### Conflict of interest

The authors declare that there is no conflict of interest.

### Authorship

AAA contributed to methodology, data characterization of the microbiology part, writing the original draft, results, discussion. WMA contributed to methodology, data characterization of the physical part, writing the original draft, results, and discussion. WKA and NHA contributed to writing the introduction. JH contributed to writing the original draft, results, and discussion.
